# Identification and Functional Analysis of Long Intergenic Non-coding RNAs Underlying Intramuscular Fat Content in Pigs

**DOI:** 10.3389/fgene.2018.00102

**Published:** 2018-03-27

**Authors:** Cheng Zou, Long Li, Xiaofang Cheng, Cencen Li, Yuhua Fu, Chengchi Fang, Changchun Li

**Affiliations:** ^1^Key Laboratory of Agricultural Animal Genetics, Breeding and Reproduction of the Ministry of Education and Key Laboratory of Swine Genetics and Breeding of the Ministry of Agriculture, Huazhong Agricultural University, Wuhan, China; ^2^The Cooperative Innovation Center for Sustainable Pig Production, Wuhan, China

**Keywords:** lincRNA, intramuscular fat content, methylation, co-expression network, pig

## Abstract

Intramuscular fat (IMF) content is an important trait that can affect pork quality. Previous studies have identified many genes that can regulate IMF. Long intergenic non-coding RNAs (lincRNAs) are emerging as key regulators in various biological processes. However, lincRNAs related to IMF in pig are largely unknown, and the mechanisms by which they regulate IMF are yet to be elucidated. Here we reconstructed 105,687 transcripts and identified 1,032 lincRNAs in pig longissimus dorsi muscle (LDM) of four stages with different IMF contents based on published RNA-seq. These lincRNAs show typical characteristics such as shorter length and lower expression compared with protein-coding genes. Combined with methylation data, we found that both the promoter and genebody methylation of lincRNAs can negatively regulate lincRNA expression. We found that lincRNAs exhibit high correlation with their protein-coding neighbors in expression. Co-expression network analysis resulted in eight stage-specific modules, gene ontology and pathway analysis of them suggested that some lincRNAs were involved in IMF-related processes, such as fatty acid metabolism and peroxisome proliferator-activated receptor signaling pathway. Furthermore, we identified hub lincRNAs and found six of them may play important roles in IMF development. This work detailed some lincRNAs which may affect of IMF development in pig, and facilitated future research on these lincRNAs and molecular assisted breeding for pig.

## Introduction

Pigs are not only a major protein source but also important biomedical models for human metabolic diseases, such as obesity, type II diabetes, and cardiovascular diseases, because their body size and physiological/anatomical features are similar to those of humans (Robich et al., 2010). During the last decade, meat producers have started to focus more on pork quality than on quantity. IMF content, which refers to the amount of fats, including phospholipids, triglycerides, and cholesterol within muscles (Hocquette et al., 2010), is one of the important factors that affect meat quality, such as flavor and drip loss (Fernandez et al., 1999; Fiedler et al., 2003). As a polygenic trait, IMF is a complex metabolic process involving many biological processes and pathways, and determined by hyperplasia and hypertrophy of adipocytes and usually develop during the latter stages of pig development (Suzuki et al., 2005; Ovilo et al., 2014). With aging, IMF development undergoes dramatic changes, such as size and number of lipocyte, fatty acid composition, and lipid content (Cameron et al., 2000; Suzuki et al., 2005; Wood et al., 2008). Studies on the mechanisms underlying IMF development not only can promote the pig breeding selection but also facilitate the studies on human obesity and its related diseases. Although previous studies have discovered some key regulators in IMF development, such as fibroblast growth factor 21 (Wang et al., 2015), miRNA-196a/b (Liu L. et al., 2017), miR-130a (Wei et al., 2017), PU.1 antisense lncRNA (Wei et al., 2015), and protein tyrosine phosphatase non-receptor type 1 (Won et al., 2017), these studies mainly focused on protein-coding genes and microRNAs, researches about the roles of lincRNAs on IMF development are scarce.

lincRNAs are a class of intergenic transcripts that are greater than 200 nt in length and have limited protein-coding potential. Owing to the development of sequencing technology, a large number lincRNAs have been identified in many species (Guttman et al., 2010; Derrien et al., 2012; Pauli et al., 2012). Mounting evidence suggests that lincRNAs play pivotal roles in various biological processes, such as gene regulation (Khalil et al., 2009; Orom et al., 2010), stem cell pluripotency (Dinger et al., 2008; Guttman et al., 2011) and skeletal muscle development (Zhao et al., 2015; Zou et al., 2017a). Meanwhile, lincRNAs related to lipid metabolism or adipogenesis in pigs have been rarely reported, and the repertoires and functional characterization of lincRNAs for IMF development in pigs are currently unclear.

Here, using the data published in a previous study, we report the systematic identification and characterization of lincRNAs in LDM from Laiwu pigs (a kind of indigenous fatty pig bred in north China) across four different ages. The pigs have significantly different IMF contents (Wang et al., 2017). We identified 1,032 putative lincRNAs and then analyzed their genomic features and expression patterns. Association analysis of DNA methylation and expression of lincRNAs was performed, and the results revealed that methylation in the promoters and the gene bodies of lincRNA genes slightly down-regulate lincRNA expression. Then, we analyzed genes neighbored or co-expressed with these lincRNAs to assign the functionalities of the later. We also identified some hub lincRNAs, which may play important roles in IMF development. Our study not only enriches the knowledge of lincRNAs in pigs, but also provides new insights into the functional studies for lincRNAs. Our work also facilitates future studies exploring the function of lincRNAs, which may be related to lipid metabolism or adipogenesis.

## Materials and Methods

### Ethics Statement and Datasets Used in This Study

All experiments in our study were performed according to the guidelines of the Key Lab of Agriculture Animal Genetics, Breeding, and Reproduction of Ministry of Education, Animal Care and Use Committee, Wuhan, 430070, China. In this study, the sows used for RNA-seq were reared under similar environmental and feeding conditions (Wang et al., 2017). Animals were humanely sacrificed as necessary to minimize suffering (Wang et al., 2017). All samples were collected from the LDM at the third lumbar (Wang et al., 2017). 12 RNA-seq datasets which included four development stages (60, 120, 240, and 400 days of age, three replicates) and two RRBS datasets (genomic DNA was pooled with an equal amount from three 120 and 240 days old pigs, respectively) we used were downloaded from the NCBI Gene Expression Omnibus (GEO) databases with accession number offered by Wang et al. (2017) (**Table 1**). The pig gene annotations were downloaded from ftp://ftp.ensembl.org/pub/release-91/gtf/sus_scrofa. Moreover, the non-redundant reference sequence (RefSeq) NR database was downloaded from ftp://ftp.ncbi.nih.gov/blast/db/.

**Table 1:** Summary of data from RNA-seq and RRBS.

	Sample	Accession number	Clean reads	Mapping ratio %	Uniquely mapping ratio %
RNA-seq data	60d_1	SRR5043824	33,532,956	83.7	73.3
	60d_2	SRR5043825	39,230,000	83.7	73.5
	60d_3	SRR5043826	36,445,032	83.4	73.8
	120_1	SRR5043827	34,665,986	83.6	74.1
	120_2	SRR5043828	34,780,852	81.5	71.9
	120_3	SRR5043829	33,425,342	83.1	73.4
	240d_1	SRR5043821	33,403,006	81.4	70.9
	240d_2	SRR5043822	34,412,034	83.7	74.4
	240d_3	SRR5043823	34,893,280	82.9	73.1
	400d_1	SRR5043818	42,825,118	82.1	71.7
	400d_2	SRR5043819	36,291,654	81.6	71.0
	400d_3	SRR5043820	36,313,474	82.7	72.5
RRBS data	120d	SRR5171452	44,613,858	68.8	63.9
	240d	SRR5171451	43,556,079	69.9	64.5


*“60d” represents 60 days, similar as “120d,” “240d,” and “400d.”*

### Transcriptome Assembly and lincRNA Identification

**FIGURE 1:**
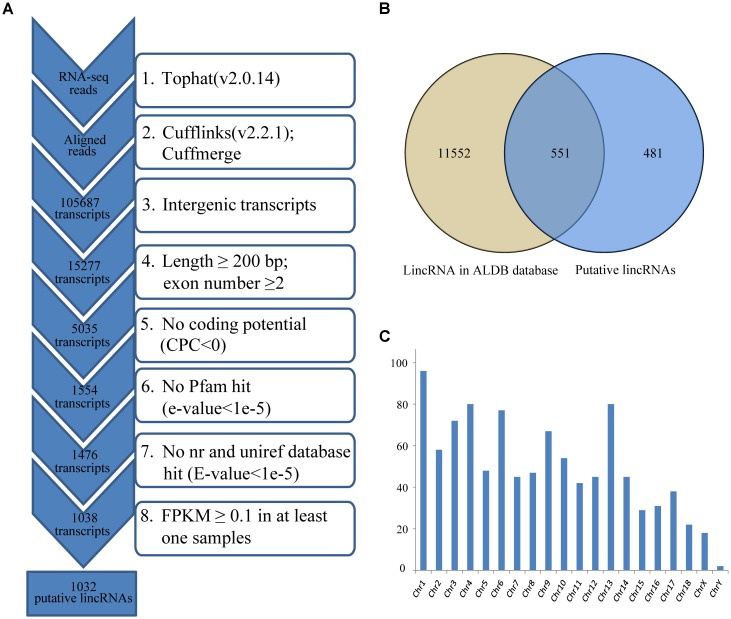
**(A)** Integrative pipeline for the identification of putative lincRNAs in this study; **(B)** Venn diagram of known and novel lincRNAs; **(C)** The chromosome distribution of putative lincRNAs. CPC, coding potential calculator; nr, non-redundant.

Reads were mapped to the pig reference genome (*Sus scrofa* 11.1)^1^ by Tophat v2.0.14 with default parameters (Trapnell et al., 2009). Then, the mapped reads were assembled through Cufflinks v2.2.1 with default parameters (and ‘min-frags-per-transfrag = 3’) (Trapnell et al., 2010; Tang et al., 2017). Meanwhile, we set the “-g” option of Cufflinks for novel transcript assembly. 12 assembled transcript files (GTF format) of four groups were then merged into a non-redundant transcriptome using Cuffmerge. And the non-redundant transcriptome was then filtered to get the putative lincRNAs. Our pipeline for lincRNA identification as shown in **Figure 1A** was based on the way described in our previous study (Zou et al., 2017b).

### Differentially Expressed lincRNAs and mRNA Analysis

Gene expression levels were estimated based on FPKM obtained by Cufflinks. We used Cuffdiff to conduct differential expression tests between two groups. A transcript will be identified differentially expressed between two groups if the absolute value of log_2_ (Fold-Change) ≥ 1 and FDR-adjusted *p*-value less than 0.05 (Benjamini et al., 2001).

### Analysis of DNA Methylation of lincRNA Genes

The RRBS data were first aligned to the pig reference genome (*Sus scrofa* 11.1) by Bismark v0.16.1 (Krueger and Andrews, 2011) with default parameters. Methylation status was determined using the bismark_methylation_extractor script provided by Bismark. The methylation percentage of each individual cytosine was calculated based on the number of methylated and unmethylated sites by bismark2bedgraph script provided by Bismark. We calculated the methylation level of the promoter and genebody region of lincRNA genes by BEDTools 2.17.0 (Quinlan and Hall, 2010) and Python scripts. We defined the promoter region as the upstream 5 kb of the transcription start site of lincRNA genes.

### Neighboring Gene Analysis

For each lincRNA locus, the nearest protein-coding genes that were transcribed nearby (<100 kb) was identified by BEDTools 2.17.0 (Quinlan and Hall, 2010). Pearson correlation of two neighbors was calculated based on their FPKM by R script.

### Weighted Gene Co-expression Network Analysis

Using the R package WGCNA (Langfelder and Horvath, 2008), we performed a WGCNA on three parts of genes (putative lincRNAs, differentially expressed protein-coding genes and protein-coding genes with expression variance ranked in the top 3000 of the data). First, a signed weighted correlation network was constructed by creating a matrix of pairwise Pearson correlation coefficients. The power of 14 was the soft-threshold and made the adjacency network exhibit scale-free topology. Next, we calculated the topological overlap matrix based on the adjacency matrix. We clustered genes into distinct modules using hierarchical clustering followed by dynamic tree cutting. We retrieved the protein-coding genes that co-expressed with lincRNAs in each module, then GO enrichment and pathway analysis were performed on them. The minimum module size was set to 30 to ensure a qualified number of genes for further analysis.

For each module, we defined the first principle component as the eigengene according to WGCNA terminology. To detect the relationship between modules and four development stages, we defined a vector to encode four development stages (Tang et al., 2017). Then, we correlated this vector with the eigengenes of each module, and a higher correlation indicated that the module was related to corresponding development stage.

### Identification of Hub lincRNAs

In four selected modules, we calculated the connectivity of each gene based on their intramodular connectivity (Langfelder and Horvath, 2008). lincRNAs with top 10% intramodular connectivity were defined as hub lincRNAs. Cytoscape_v3.5.0 software was used for network visualization (Shannon et al., 2003; Yang et al., 2014).

### Gene Ontology and Pathway Analysis

We performed DAVID analysis by running queries for each protein-coding gene against the DAVID database (Huang da et al., 2009). Because the annotation for the genes in *Sus scrofa* is relatively limited, all genes were firstly converted into human homologous genes using BIOMART from Ensembl.^2^

### Quantification of lincRNAs Through qRT-PCR

Total RNA was extracted from each tissue by using TRIzol reagent (Invitrogen), in accordance with the manufacturer’s protocol. The concentration and quality of RNA were confirmed using the Agilent 2100 system. We calculated the expression level of ten hub lincRNAs in eight tissues, and each lincRNA had three technical repeats. Ten pairs of primers for qRT-PCR were designed using the Oligo 7 program (Supplementary Table S7). The 18s rRNA served as the endogenous control gene. QRT-PCR was performed with SYBR Green (Bio-Rad) to assess the expression level of each lincRNAs. Ct values were calculated using Sequence Detection System software (Applied Biosystems), and the amount of target sequence normalized to the reference sequence was calculated as 2^-ΔΔ*C*_t_^.

## Results

### Identification and Characterization of lincRNAs

We used RNA-seq data from published study including four groups, which had different IMF contents (Wang et al., 2017), to identify lincRNAs in LDM related to IMF development. Approximately 356.1 of 430.2 million clean reads were mapped to the whole genome of *Sus scrofa* (11.1) (**Table 1**). Then, we reconstructed a transcriptome for each sample through Cufflinks, and all transcripts were pooled into a unique merged transcript set through Cuffmerge (Trapnell et al., 2012). We obtained 105,687 transcripts, of which 15,277 were intergenic transcripts. We identified lincRNAs from the 15,277 transcripts according to the pipeline shown in **Figure 1A**. Finally, we obtained 1,032 putative lincRNAs originating from 712 gene loci and 551 of these lincRNAs had no overlaps with currently annotated coding or non-coding transcripts (**Figure 1B**, Supplementary Data Sheet S1 and Supplementary Table S1). These putative lincRNAs were distributed in all chromosomes (**Figure 1C**).

**FIGURE 2:**
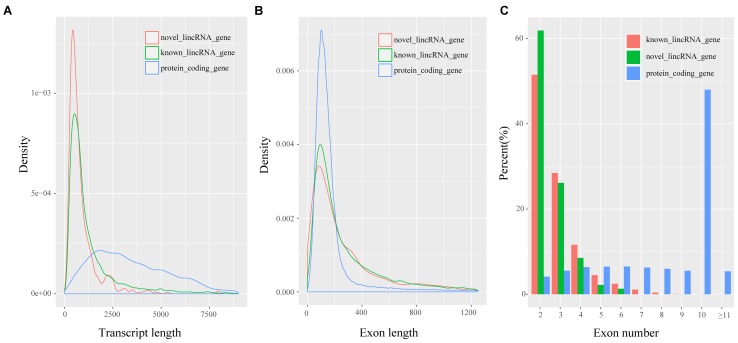
Characteristics of putative lincRNAs. **(A)** Comparison of transcript size distribution; **(B)** Comparison of exon size distribution; **(C)** Comparison of exon number.

Previous studies showed many differences, particularly in transcript length and exon number, between lincRNAs and protein-coding transcripts (Derrien et al., 2012; Liu S. et al., 2017). We analyzed the features of novel lincRNAs and compared them with those of protein-coding transcripts and known lincRNAs according to the reconstructed transcriptomes. A total of 45,775 protein-coding transcripts corresponding to 22,329 genes in the pig annotation in Ensembl database, and 12,103 known lincRNA transcripts encoded by 7,381 lincRNA genes in the pig lincRNA annotation in the domestic-animal lncRNA database (ALDB) exist (Zhou et al., 2014; Li et al., 2015). We used the Kolomogorv–Smirnov-test method to test the differences. We found that overall novel lincRNA transcripts (mean 872 bp) were shorter than known lincRNA transcripts (mean 1,362 bp; *p*-value < 2.2e-16) and protein-coding transcripts (mean 3,483 bp; *p*-value < 2.2e-16) (**Figure 2A**). Meanwhile, the average exon length of lincRNA transcripts was 342 bp. This value was shorter than that of known lincRNA transcripts (480 bp; *p*-value < 2.2e-16) but longer than that of protein-coding transcripts (291 bp; *p*-value < 4.2e-4) (**Figure 2B**). Furthermore, the average exon number of the novel lincRNA transcripts (2.5) was similar to that of the known lincRNA transcripts (2.8; *p*-value < 6.5e-12) but fewer than that of the protein-coding transcripts (11.9; *p*-value < 2.2e-16) (**Figure 2C**). Features, such as shorter transcript length, longer exon length and fewer exon number of lincRNAs compared with protein-coding genes were consistent with previous reports (Li et al., 2016; Tang et al., 2017).

### Expression Analysis of lincRNAs

**FIGURE 3:**
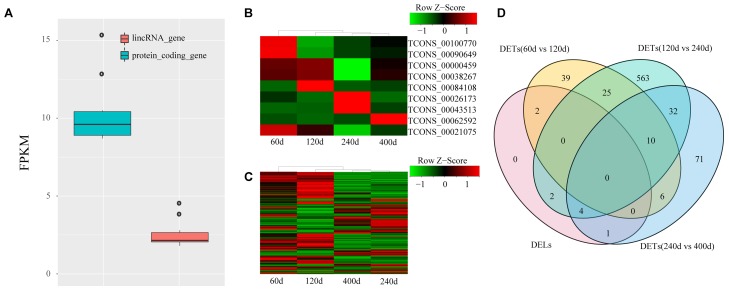
Expression profile of lincRNAs. **(A)** Comparison of expression level between lincRNAs and protein-coding genes; **(B)** Heat map of nine DELs among four groups; **(C)** Heat map of 746 DETs among four groups. Red, increased expression; black, neutral expression; green, decreased expression; **(D)** Venn diagram of DELs and DETs among four groups. “60d” represents 60 days, similar as “120d,” “240d,” and “400d.”

lincRNAs tend to have lower expression level compared with protein-coding transcripts (Billerey et al., 2014; Li et al., 2016). We compared the FPKM of the 1,032 lincRNAs to that of protein-encoding transcripts to explore the expression profile of the lincRNAs we identified. We also found that the putative lincRNAs showed significantly lower expression than the protein-coding transcripts (2.6 FPKM vs. 10.3 FPKM; Kolomogorv–Smirnov test, *p*-value < 2.2e-16) (**Figure 3A**). Then, we conducted differential expression analysis between adjacent development stages (60 days vs. 120 days; 120 days vs. 240 days; 240 days vs. 400 days) by Cuffdiff. Finally, we identified 755 DETs including nine DELs (**Figures 3B–D** and Supplementary Table S2). The comparison between 120 and 240 days comprised the majority of DETs (**Figure 3D**).

### Methylation Analysis of lincRNAs

**FIGURE 4:**
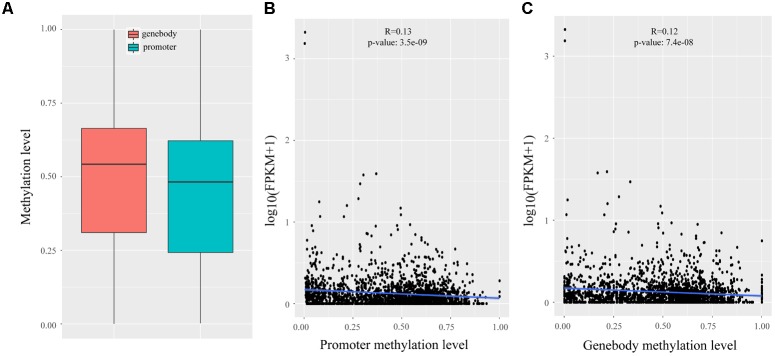
Methylation analysis of lincRNAs. **(A)** Comparison of methylation level between the promoter and genebody of lincRNAs; **(B)** Scatter plot of the methylation level of lincRNA promoter and lincRNA expression; **(C)** Scatter plot of the methylation level of lincRNA genebody and lincRNA expression. The Pearson correlation coefficient was calculated between the log2 ratios of lincRNA expression and lincRNA methylation. The line represented regression line. The statistical significance was calculated by R language (version: 3.3.3).

In Wang’s study, reduced representation bisulfite sequencing (RRBS) was performed on two development stages that had the largest difference in IMF (120 days: 3.59%; 240 days: 9.88%) (Wang et al., 2017), which enabled us to analyze the methylation statues of lincRNAs. We mapped the RRBS data to the pig reference genome and calculated the methylation level of lincRNAs by Bismark (Krueger and Andrews, 2011). The methylation level of lincRNA promoter and genebody were compared, and the results showed that lincRNA genebody had significantly higher methylation level than the lincRNA promoter (two sample *t*-test, *p*-value < 1.4e-11) (**Figure 4A**). This result was similar to those of previous studies on lincRNAs and protein-coding genes (Sati et al., 2012; Huang et al., 2014; Yang et al., 2017a; Zou et al., 2017a). Many studies proved that DNA methylation can regulate gene expression (Ball et al., 2009; Laurent et al., 2010; Schachtschneider et al., 2015), so we determined whether any regulatory relationship exists between lincRNA methylation and their expression. Association analysis of methylation and expression of lincRNAs revealed that methylation in both promoter and genebody of lincRNAs can significantly down-regulate its expression (*p*-value < 3.5e-9, *p*-value < 7.4e-8; **Figures 4B,C**).

### Nearest Neighbor Analysis of lincRNAs

**FIGURE 5:**
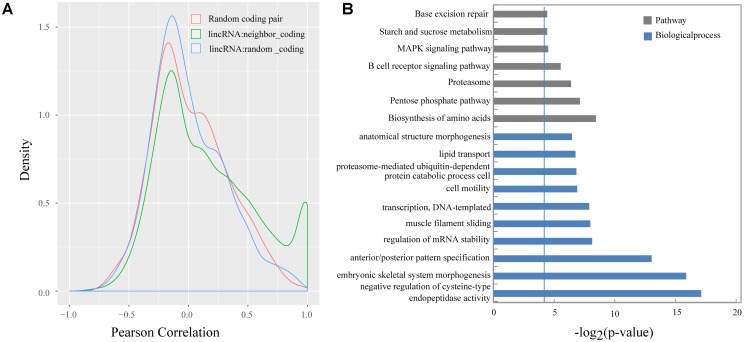
**(A)** Density histograms of Pearson correlation coefficient between genes of different classes; **(B)** Gene ontology and pathway analysis of protein-coding genes next to lincRNAs. The vertical line represents the threshold of significance [–log_2_(0.05)≈4.3].

Previous studies demonstrated that lincRNAs can affect the gene expression of their neighbors (Ponjavic et al., 2009; Orom et al., 2010; Casero et al., 2015). We calculated the Pearson correlation coefficient between the expression of lincRNAs and their protein-coding neighbors to explore the expression relationship between lincRNAs and their neighboring protein-coding genes. Our results suggested that lincRNAs exhibited a stronger correlation with their neighbors (mean 0.16) compared with randomly selected protein-coding genes (mean 0.0066), and also were stronger than randomly selected protein-coding gene pairs (mean 0.019) (Kolomogorv–Smirnov test, both *p*-value < 2.2e-16) (**Figure 5A**). Our results were consistent with Liu’s and Xia’s study (Li et al., 2016; Wu et al., 2016).

Several studies indicated that some lincRNAs may act in *cis* to regulate their neighbors, therefore those protein-coding genes, which are transcribed nearby (<100 kb) the lincRNAs, may contribute to interpreting the lincRNA function (Wang et al., 2011; Zhao et al., 2015). In total, we obtain 2,571 protein-coding neighbors (Supplementary Table S3). Then, we performed DAVID analysis by running queries for each protein-coding gene against the DAVID database. The DAVID results suggested that 620 of the 2,571 protein-coding neighbors significantly participated in 48 biological processes and 7 pathways. Of them, 115 protein-coding neighbors participated in some lipid-metabolism-related biological processes or pathways, such as lipid transport, cholesterol homeostasis, and MAPK signaling pathway (**Figure 5B** and Supplementary Table S4).

### Function Prediction of lincRNAs

**FIGURE 6:**
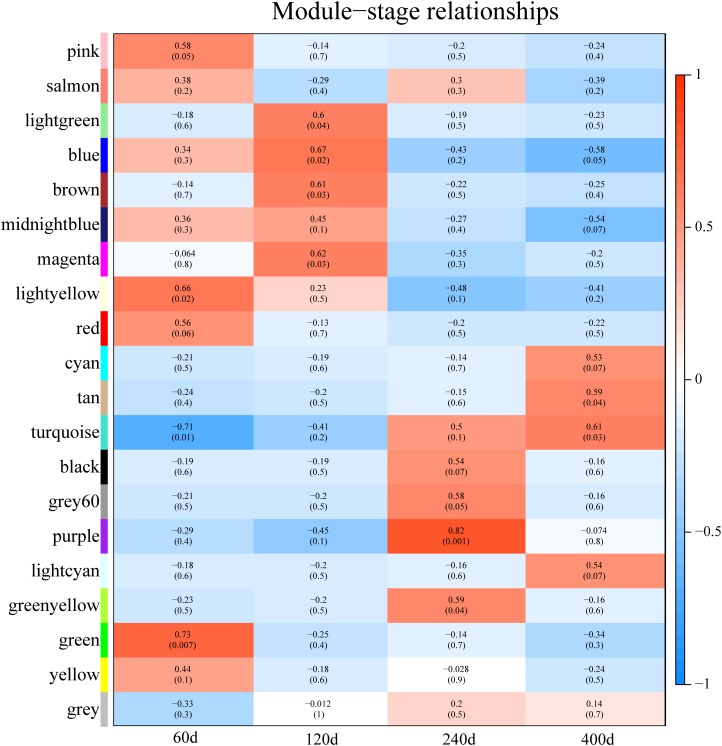
Module-stage correlations and corresponding *p*-values. On the left, different colors represent different modules. Each cell contains the correlation and *p*-value given in parentheses. Cells are color-codes by the correlation according to the color legend on the right. Red, positive correlation; white, none correlation; blue, negative correlation. “60d” represents 60 days, similar as “120d,” “240d,” and “400d.”

Predicting putative function of lincRNAs is still challenging because of the lack of annotation and low expression level. In previous studies, correlation-based approaches were used to infer the function of lincRNAs (Liao et al., 2011; Ramos et al., 2013; Casero et al., 2015). Here, we constructed a co-expression network to associate lincRNAs with protein-coding genes by performing WGCNA (Langfelder and Horvath, 2008). Though the clustering of correlated genes 20 distinct modules with module size ranging from 75 to 817 (mean: 231; median: 181) were obtained (Supplementary Table S5). We carried out the correlation analysis between modules and different development stages and found that eight modules were strongly associated with different development stages (correlation ≥ 0.6, *p*-value < 0.05) (**Figure 6**), possibly representing key gene networks operating in each stage. Notably, seven of the eight modules exhibited a positive correlation with one development stages and the turquoise module was significantly related with two development stages (60 and 400 days).

**FIGURE 7:**
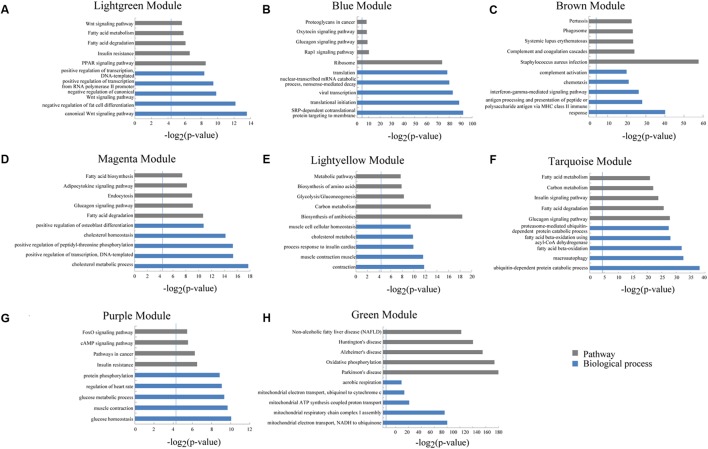
Gene ontology and pathway analysis of protein-coding genes in different modules. **(A–H)** Gene ontology and pathway analysis results of protein-coding genes in eight modules. The vertical line represents the threshold of significance [-log_2_(0.05)≈4.3].

**FIGURE 8:**
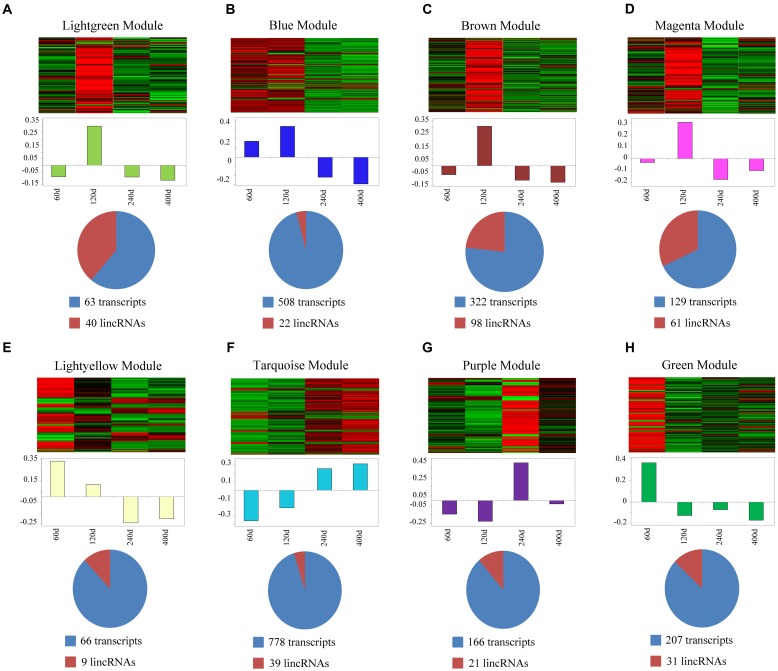
Co-expression networks of lincRNAs and protein-coding genes in different modules. **(A–H)** Top of each panel: heat maps of expression level for eight modules of co-expressed genes in four stages. Red, increased expression; black, neutral expression; green, decreased expression. Middle of each panel: bar plots of the values of corresponding module eigengenes. Bottom of each panel: pie charts showing the abundance of lincRNAs within each module. “60d” represents 60 days, similar as “120d,” “240d,” and “400d.”

We used a method termed “guilt by association” reported in previous studies to infer the potential function of putative lincRNAs (Guttman et al., 2009; Rinn and Chang, 2012). For the aforementioned eight modules, we performed functional annotation and enrichment analysis on protein-coding genes in each module (**Figures 7A–H**). We found that genes in the four modules (lightgreen, magenta, lightyellow and turquoise) remarkably participated in IMF-related biological processes or pathways, such as fatty acid metabolism and cholesterol metabolic and adipocytokine signaling pathways (**Figures 7A,D–F** and Supplementary Table S6). This finding suggested that lincRNAs in these modules may play vital roles in IMF development. Furthermore, genes in the lightgreen and magenta modules were up-regulated in 120 days; in the lightyellow module were up-regulated in 60 days. Meanwhile, genes in the turquoise module were down-regulated in 60 days but up-regulated in 400 days (**Figure 8**).

### Identification of Hub lincRNAs

**FIGURE 9:**
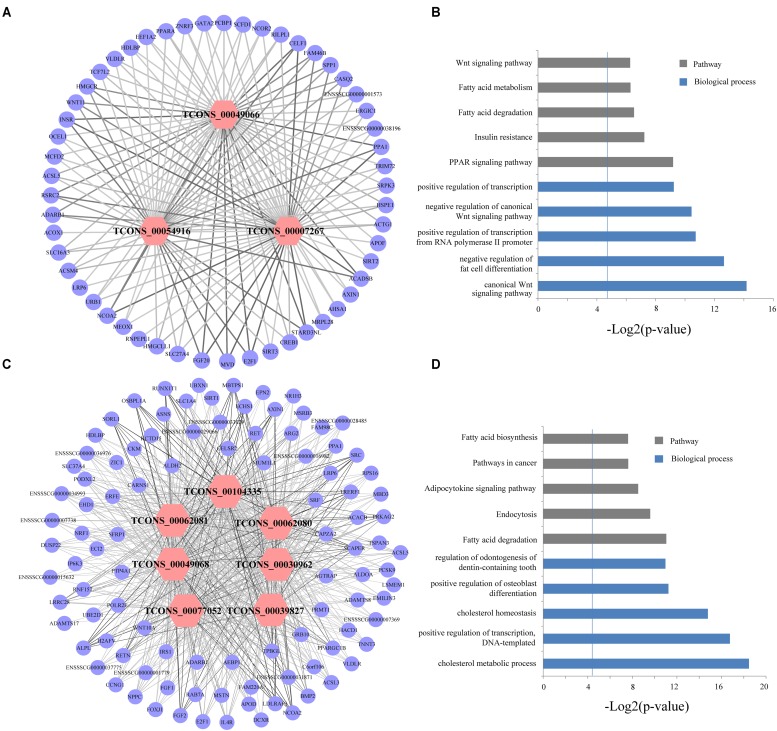
**(A,C)** Network visualization of the co-expression of hub lincRNAs and protein-coding genes in lightgreen and magenta module, respectively. Outer circles indicate protein-coding genes; inner hexagons indicate hub lincRNAs. The line thickness represents the correlation between lincRNAs and protein-coding genes. **(B,D)** Gene ontology and pathway analysis of protein-coding genes co-expressed with hub lincRNAs in lightgreen and magenta module, respectively. The vertical line represents the threshold of significance (-log_2_(0.05)≈4.3).

**FIGURE 10:**
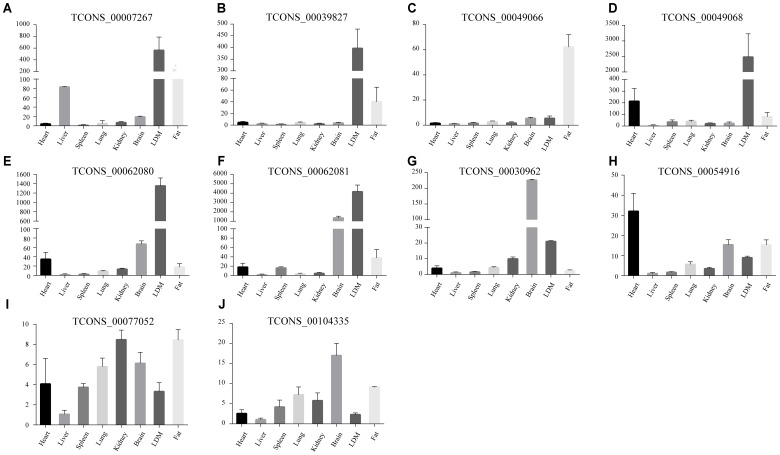
**(A–J)** The expression of ten hub lincRNAs in eight tissues. The *Y* axis represents relative expression level. Results are presented as mean values ± SEM.

Abovementioned results revealed that genes in the four modules (lightgreen, lightyellow, magenta, and turquoise) may exhibit important functions in IMF development. Thus we selected these four modules for further hub lincRNA analysis. Hub genes are centrally located in a scale-free network of each module and reflect the core functions of the network (Horvath and Dong, 2008). We measured the intramodular connectivity (also named weight) of each gene by WGCNA to identify hub lincRNAs in each module. In the four modules, we detected three and seven hub lincRNAs in lightgreen and magenta module, respectively. However, no hub lincRNA was detected in lightyellow and turquoise modules. Besides, we found that two DELs (*TCONS_00000459* and *TCONS_00084108*) were in the magenta module, but none of them was identified as hub lincRNAs. We constructed two correlation networks between these hub lincRNAs and protein-coding genes, which co-expressed with them by Cytoscape (Shannon et al., 2003), to further clarify the function of hub lincRNAs in two modules (**Figures 9A,C**). Then, we performed gene ontology and pathway analysis on protein-coding genes that co-expressed with hub lincRNAs. The DAVID results indicated that these protein-coding genes significantly enriched in IMF-related biological processes or pathways including peroxisome proliferator-activated receptor signaling pathway, negative regulation of fat cell differentiation, and fatty acid degradation, indicating an important effect of these hub lincRNAs on IMF (**Figures 9B,D**). Moreover, we examined the expression profile of 10 hub lincRNAs in eight tissues through quantitative reverse transcription polymerase chain reaction (qRT-PCR), and found that six hub lincRNAs (*TCONS_00007267*, *TCONS_00039827*, *TCONS_00049066*, *TCONS_00049068*, *TCONS_00062080*, and *TCONS_00062081*) were highly expressed in the muscle or fat (**Figure 10**). Although knowledge about how these hub lincRNAs involved in IMF development are quite limited, these lincRNAs would serve as ideal candidates for further functional studies.

## Discussion

Large-scale genomic studies revealed that mammalian genomes are populated with a substantial portion of long non-coding RNAs, and majority of them are lincRNAs (Carninci et al., 2005; Guttman et al., 2010; Cabili et al., 2011). Although some lincRNAs have been reported in pigs (Zhou et al., 2014; Zhao et al., 2015; Li et al., 2016; Tang et al., 2017; Yang et al., 2017b; Yu et al., 2017) and a few lincRNAs have been suggested to play a regulatory role in various developmental contexts, numerous lincRNAs are still not identified compared with humans and mice. Especially, several lincRNAs related to IMF development in pigs remain unidentified and uncharacterized. Here, we present the comprehensive identification and analysis of lincRNAs in pig LDM based on published RNA-seq data (Wang et al., 2017). The 1,032 putative lincRNAs in our study not only enrich the pig lincRNA annotation, but also facilitate future studies on pig lincRNAs. Previous studies revealed that lincRNAs exhibit high tissue specificity (Cabili et al., 2011; Pauli et al., 2012; Tang et al., 2017). Thus, a part of these lincRNAs may specifically express in LDM and exert some functions in muscle development. Although muscle is a major metabolic tissue in pigs and involved in diverse biological processes, such as muscle growth and lipid metabolism, we provided considerable attention to lincRNAs potentially related to IMF development in this study.

We characterized the putative lincRNAs in our study and found that they shared some similar features (smaller size, fewer exon number and lower expression) compared with protein-coding genes with lincRNAs reported in previous studies (Pauli et al., 2012; Li et al., 2016; Liu S. et al., 2017). Here, we obtained very few DELs (nine) between adjacent stages, but we found that the ratio of different expression levels of lincRNAs was approximate with that of protein-coding genes (9/1,032 or 0.87%; 746/104,655 or 0.71%). We found that the overall methylation of both promoter and genebody of lincRNAs can down-regulate its expression. In previous study, Zhang reported that lincRNA genes always have higher methylation levels in promoter and genebody than that of protein-coding genes (Zhou et al., 2015). Therefore, we conjectured that higher methylation level in lincRNA genes may contribute to their low expression compared with protein-coding genes.

In the present study, we identified some lincRNAs, which may exert some functions in muscle. However, in contrast to the substantial progress in lincRNA discovery, the most challenging obstacle in lincRNA analysis is the determination of their biological functions. Here, we predicted the function of lincRNAs based on the analysis of protein-coding genes which neighbored or co-expressed with these lincRNAs. Several studies revealed that lincRNAs may act in *cis* to regulate the expression of neighboring protein-coding genes (Wang et al., 2011; Zhang et al., 2014). We found that lincRNAs exhibited a strong correlation with their neighbors, and some of their neighbors participated in lipid transport and MAPK signaling pathway. Thus, we speculated that some lincRNAs may participate in IMF development by regulating their protein-coding neighbors. However, the mechanism by which individual lincRNA regulates its neighbors is worth of further research. We constructed a co-expression network by WGCNA to clearly understand the functional roles of lincRNAs. We selected four modules, wherein protein-coding genes were significantly involved in IMF-related biological processes or pathways for further analysis. Previous studies suggested that genes co-expressed in the same module may have similar biological functions (Guttman et al., 2009; Rinn and Chang, 2012; Marques and Ponting, 2014). Thus lincRNAs in these four modules were inferred to be functionally related with IMF development. Moreover, we found that genes in three modules (lightgreen, magenta and lightyellow) were up-regulated in early development of IMF (60 and 120 days), indicating that genes in these three modules may mainly exert their functions in early IMF deposition, whereas genes in the turquoise module were down-regulated in early development (60 and 120 days) but up-regulated in later IMF development (240 and 400 days). Considering that some genes in the turquoise module were enriched in pathways, such as fatty acid beta-oxidation by using acyl-CoA dehydrogenase and fatty acid degradation, we inferred that genes in turquoise module may promote fatty consumption.

We identified 10 hub lincRNAs as aforementioned and found that six of them were especially expressed in LDM or fat. This result indicated their potential important role in LDM and fat. Besides, based on the hub lincRNA networks, we found that six tissue-specially expressed hub lincRNAs exhibited high correlation with two protein-coding genes (nuclear receptor coactivator 2, *NCOA2*; E2F transcription factor 1, *E2F1*) (weight ranged from 0.55 to 0.71). Previous studies demonstrated that *NCOA2* and *E2F1* are involved in many IMF-related biological processes, such as oxidative metabolism, lipid metabolism and adipogenesis (Blanchet et al., 2011; Hartig et al., 2011; Denechaud et al., 2016). Loewer and Durruthy revealed that lincRNAs can exert their functions through related protein-coding genes (Loewer et al., 2010; Durruthy-Durruthy et al., 2016). Together, these results indicated that six tissue-specially expressed hub lincRNAs might play crucial roles in IMF development.

In previous study, we found some lincRNAs in LDM may participate in some fat-deposition-related biological process or pathways, such as fatty acid degradation and adipocytokine signaling pathway, and contributed to meat quality differences between lean-type and fat-type pigs by co-expressing with their potential target genes (Zou et al., 2017b). Besides, in Yu’s study, they identified 794 potential target genes of differentially expressed lncRNAs involved in adipocytokine signaling pathways and calcium signaling pathways between lean and obese pigs (Yu et al., 2017). They also found that some differentially expressed lncRNAs were in quantitative trait loci, which are associated with fat-deposition-related traits, such as abdominal fat weight and average backfat thickness (Yu et al., 2017). The association analysis between lincRNAs and quantitative trait loci in Yu’s study provided new insights into our following work (Yu et al., 2017). For next step, lincRNAs that potentially be related to fat-deposition in these studies will be reanalyzed and functionally validated, and lincRNAs in the aforementioned four modules especially the six tissue-specially expressed hub lincRNAs in the present study will be our priority.

In summary, we identified and characterized a number of lincRNAs involved in IMF development. Functional analysis revealed that many lincRNAs were involved in IMF-related processes. Given that the role of lincRNAs in pigs has not been fully identified and annotated, this work provides a valuable resource for further studies and helps understand the potential functions of lincRNAs in IMF development. The hub lincRNAs represent ideal candidates for further researches about lipid metabolism, and further experimental exploration of these lincRNAs will be our next step.

## Author Contributions

ChL conceived and designed the experiments and explained the data. CZ analyzed the main content of the data with the help of CeL, YF, and CF. LL performed the experiment with the help of XC. CZ wrote the paper with the help of ChL.

## Conflict of Interest Statement

The authors declare that the research was conducted in the absence of any commercial or financial relationships that could be construed as a potential conflict of interest.

## Footnotes

1 ftp://ftp.ensembl.org/pub/release-91/fasta/sus_scrofa/dna/

2 http://asia.ensembl.org/biomart/martview/265b18e4080d32cc7326dc26cfc3bdf0

## Abbreviations

DAVID: database for annotation, visualization and integrated discovery
DELs: differentially expressed lincRNAs
DETs: differentially expressed transcripts
FPKM: fragments per kilobase of transcript per million mapped reads
IMF: intramuscular fat
LDM: longissimus dorsi muscle
lincRNAs: long intergenic non-coding RNAs
WGCNA: weighted gene co-expression network analysis
